# Continuous Measurement of Radial Free Forearm Flap Tissue Perfusion for Flap Monitoring After Microvascular Head and Neck Reconstruction—Systemic Blood Pressure as a Potential Confounder in the Early Postoperative Period

**DOI:** 10.3390/jcm14082561

**Published:** 2025-04-08

**Authors:** Mark Ooms, Philipp Winnand, Marius Heitzer, Nils Vohl, Anna Bock, Johannes Bickenbach, Frank Hölzle, Ali Modabber

**Affiliations:** 1 Department of Oral and Maxillofacial Surgery, University Hospital RWTH Aachen, Pauwelsstraße 30, 52074 Aachen, Germany; 2 Department of Intensive Care Medicine, University Hospital RWTH Aachen, Pauwelsstraße 30, 52074 Aachen, Germany

**Keywords:** microsurgery, free flap, flap perfusion monitoring, blood pressure, blood flow, oxygen saturation

## Abstract

**Background:** Continuous measurement of flap tissue perfusion in the context of postoperative flap monitoring after microvascular head and neck reconstruction may be confounded by alterations in systemic blood pressure, particularly when using predefined absolute thresholds for the detection of vascular flap compromise. This study aimed to investigate the relationship between radial free forearm flap (RFFF) tissue perfusion and systemic blood pressure following head and neck reconstruction. **Methods:** The study included 14 patients reconstructed with an RFFF in the head and neck region between 2020 and 2022. Eight hours of postoperatively recorded flap tissue perfusion, determined with an attached surface probe at a 3 mm tissue depth with the Oxygen-2-See (O2C) analysis system, in terms of blood flow and hemoglobin oxygen saturation, and systemic blood pressure, determined as absolute and relative values (difference between measured and preoperative values), in terms of systolic blood pressure (SBP), diastolic blood pressure (DBP), and mean arterial blood pressure (MBP), were analyzed for correlations. **Results:** Blood flow and hemoglobin oxygen saturation were generally indifferent between the hourly measurement intervals. Blood flow was correlated with relative DBP and MBP values (0.191, *p* < 0.001; and 0.213, *p* < 0.001). These correlations persisted upon controlling for norepinephrine, propofol, or sulfentanyl (all *p* < 0.001). **Conclusions:** Early postoperative RFFF tissue perfusion measured with attached surface probes for the O2C analysis system remains constant but correlates with systemic blood pressure in terms of blood flow and relative blood pressure values. This highlights the importance of maintaining constant systemic blood pressure during RFFF tissue perfusion measurement for postoperative flap monitoring.

## 1. Introduction

Reconstruction of the head and neck region, for example, after the resection of a malignant disease, is routinely performed using microvascular free flaps to restore both functionality and aesthetics, with the radial free forearm flap (RFFF) being widely used [1,2]. However, flap failure due to vascular compromise occurs, with detrimental consequences for the patient, such as permanent functional impairment and aesthetic disfigurement [3,4].

Postoperative flap monitoring is crucial in microvascular head and neck reconstruction to timely detect vascular flap compromise, as adequate tissue perfusion based on sufficient arterial blood inflow and venous blood outflow through patent anastomosis is a prerequisite for flap viability and, thus, flap survival, and a prolonged interval of insufficient flap tissue perfusion between the onset and correction of vascular flap compromise significantly reduces the chance of flap salvage [5,6,7,8,9,10]. Due to the limitations of clinical flap monitoring, e.g., the need for clinical experience and the delay in the clinical signs of vascular flap compromise, technical methods such as the Oxygen-2-See (O2C) analysis system are used for postoperative flap monitoring [5,7,8,9].

The O2C analysis system is based on measuring flap tissue perfusion in relation to predefined thresholds indicating vascular flap compromise, including absolute thresholds for blood flow (<60 arbitrary units [AU] for RFFFs) and hemoglobin oxygen saturation (<40% for RFFFs), with attached surface probes allowing continuous flap tissue perfusion measurement for postoperative flap monitoring [5,7,8,11]. In this context, systemic blood pressure may be a confounding factor, particularly regarding the predefined absolute thresholds, as blood pressure is, in addition to blood vessel length, blood vessel diameter, and blood viscosity, an important determinant of tissue perfusion and, thus, probably also of flap tissue perfusion [5,8,12,13,14]. However, the relationship between flap tissue perfusion and systemic blood pressure remains unknown, as studies on this topic included patients with different flap types (e.g., RFFFs, fibula free flaps, anterolateral thigh flaps, and latissimus dorsi flaps), did not provide distinct parameters of flap tissue perfusion, e.g., tissue blood flow and hemoglobin oxygen saturation, and showed conflicting results regarding confirmation or denial of an influence of systemic blood pressure on flap tissue perfusion, contributing to the existing lack of knowledge about the physiology of microvascular free flap perfusion and the need to adjust absolute thresholds for flap monitoring based on flap tissue perfusion with the O2C analysis system [15,16,17].

The aim of this study was to investigate the relationship between RFFF tissue perfusion and systemic blood pressure in the context of postoperative flap monitoring after microvascular head and neck reconstruction in the early postoperative period.

## 2. Materials and Methods

### 2.1. Study Population

The retrospective study was conducted according to the guidelines of the Declaration of Helsinki and approved by the local ethics committee of the Medical Faculty RWTH Aachen University (EK 22-358).

The study population consisted of 14 patients who underwent microvascular reconstruction in the head and neck region using an RFFF after the resection of malignant disease (squamous cell carcinoma (n = 10), sarcoma (n = 1), Merkel cell carcinoma (n = 1), adenocarcinoma (n = 1), and adenoid cystic carcinoma (n = 1)) in our Department of Oral and Maxillofacial Surgery between 2020 and 2022 (11 November 2020 until 27 April 2022). The inclusion criteria were a continuous flap tissue perfusion measurement, age over 18 years, and the availability of complete data sets.

The data were extracted from medical records. Surgery duration was defined as the time interval between the first incision and the last suture, and flap ischemia duration was defined as the time interval between cessation of flap perfusion at the donor site after transection of the flap pedicle and restoration of flap perfusion at the recipient site after release of the microsurgical anastomosis.

Surgical procedures were performed under general anesthesia. The flap locations were intraoral, including the tongue, the floor of the mouth, the mandible after resection of the lower gingiva and bone continuity resection with placement of a reconstruction plate, or the cheek, and extraoral, including the buccal region, the infraorbital region, or the orbital region. The arterial anastomosis was conducted between the cervical recipient vessel (facial artery, lingual artery, or superior thyroid artery) and the flap pedicle vessel in an end-to-end configuration and the venous anastomosis was conducted between the cervical recipient vessel (internal jugular vein, internal jugular vein + anterior jugular vein, internal jugular vein + vena comitans of hypoglossal nerve, facial vein, or retromandibular vein) in an end-to-end or end-to-side configuration. Patients were monitored postoperatively in the intensive care unit under mechanical ventilation and analgosedation at least until the next morning.

### 2.2. Blood Pressure Measurement Data

The data for continuous systemic blood pressure measurement during the postoperative period of the first eight hours were taken from the IntelliSpace Critical Care and Anesthesia ICCA data management system (Philips Medizin Systeme Boeblingen GmbH, Boeblingen, Germany; Software version J.05.01).

The absolute blood pressure values for systolic blood pressure (SBP) and diastolic blood pressure (DBP) were measured invasively with an arterial catheter at five-minute intervals using the IntelliVue X2 M3002A device (Philips Medizin Systeme Boeblingen GmbH, Boeblingen, Germany; Software version M.04.05), and the mean arterial blood pressure (MBP) was calculated according to the following commonly used formula: MBP = DBP + 1/3 × (SBP—DBP) [18].

The relative blood pressure values were calculated as the difference between the postoperatively measured blood pressure values and the mean value of all available preoperatively measured blood pressure values before the induction of anesthesia in the operating room.

### 2.3. Flap Perfusion Measurement Data

The data for continuous flap tissue perfusion measurement during the postoperative period of the first eight hours were extracted from the O2C analysis system (Oxygen-to-see type, LEA Medizintechnik, Giesen, Germany; Software version 43.04).

**Figure 1:**
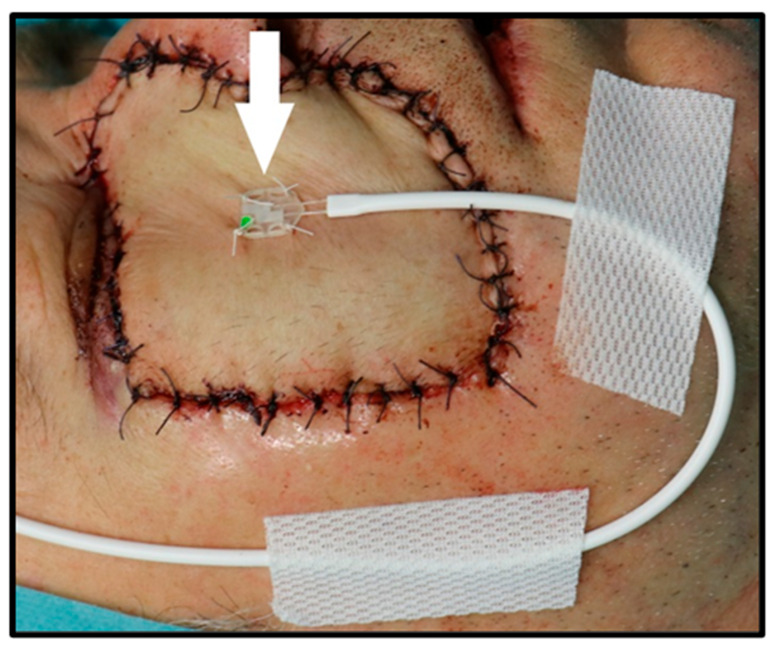
Measurement of flap perfusion. Measurement of perfusion of a radial free forearm flap with an attached surface probe (white arrow) in a patient reconstructed in the right infraorbital region.

Flap tissue perfusion values, including blood flow and hemoglobin oxygen saturation, were measured at one-minute intervals at a tissue depth of 3 mm using an attached surface probe fixed with four sutures in the middle of the RFFF (Figure 1).

The flap tissue perfusion parameter blood flow was determined by Doppler spectroscopy (830 nm; 30 mW) and calculated as the product of erythrocyte quantity (based on the analysis of the sum of light absorbances) and velocity (based on the analysis of the Doppler shift of light wave frequencies due to the movement of the erythrocytes in the blood vessels) [5,19].

The flap tissue perfusion parameter hemoglobin oxygen saturation was determined by white-light spectroscopy (500–800 nm; 50 W) as the color change of the light absorbances in comparison to prerecorded hemoglobin spectra with defined oxygen saturation [5,19].

### 2.4. Statistical Analysis

Data were presented as numbers (with percentage) or medians (with interquartile range). Data for flap tissue perfusion measurement and systemic blood pressure measurement values were analyzed as mean values over 15 min intervals (32 intervals per patient).

Differences in flap tissue perfusion measurement between time intervals (0–1 h, 1–2 h, 2–3 h, 3–4 h, 4–5 h, 5–6 h, 6–7 h, and 7–8 h) were analyzed using the Friedman test (between all time intervals; n = 448) and the Dunn–Bonferroni post hoc test (between two time intervals: 1–2 h vs. 0–1 h, 2–3 h vs. 1–2 h, 3–4 h vs. 2–3 h, 4–5 h vs. 3–4 h, 5–6 h vs. 4–5 h, 6–7 h vs. 5–6 h, and 7–8 h vs. 6–7 h; n = 56 per interval) separately for blood flow and hemoglobin oxygen saturation.

Associations between flap tissue perfusion measurement and systemic blood pressure measurement values, specifically SBP, DBP, and MBP, were analyzed by calculating the Spearman correlation coefficient (n = 448) and the partial Spearman correlation coefficient (controlling for the administered doses of norepinephrine, propofol or sulfentanyl) (n = 448) separately for blood flow and hemoglobin oxygen saturation.

Values of *p* < 0.05 were considered statistically significant. The statistical analysis was performed using SPSS version 28 (SPSS, IBM, New York, NY, USA).

## 3. Results

### 3.1. Study Population

The study population consisted of 14 patients, including five men and nine women (Table 1). The flap locations were intraoral in 10 patients (tongue in four patients, mouth floor in two patients, mandible in three patients, and cheek in one patient) and extraoral in four patients (buccal in two patients, infraorbital in one patient, and orbital in one patient).

### 3.2. Flap Tissue Perfusion Measurement Values over Time

**Figure 2:**
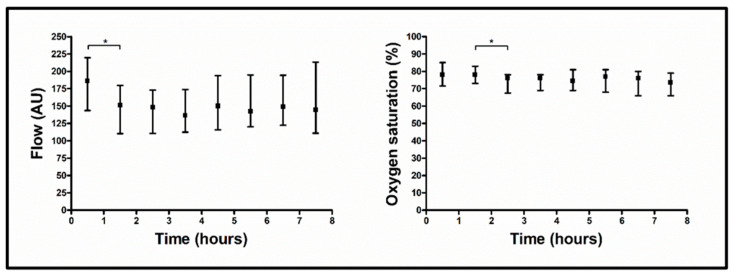
Comparison of flap perfusion measurement values over time. Data shown as median (with interquartile range) for blood flow (**left**) and hemoglobin oxygen saturation (**right**) separately for each measurement interval (0–1 h, 1–2 h, 2–3 h, 3–4 h, 4–5 h, 5–6 h, 6–7 h, and 7–8 h); *p*-values corresponding to testing for differences between measurement time intervals with Dunn-Bonferroni post hoc test (1–2 h vs. 0–1 h, 2–3 h vs. 1–2 h, 3–4 h vs. 2–3 h, 4–5 h vs. 3–4 h, 5–6 h vs. 4–5 h, 6–7 h vs. 5–6 h, and 7–8 h vs. 6–7 h) after Friedman test (blood flow *p* < 0.001; hemoglobin oxygen saturation *p* < 0.001); * *p* < 0.05; abbreviations: AU = arbitrary units.

The flap tissue perfusion varied across the time intervals in terms of blood flow (*p* < 0.001), exhibiting lower blood flow in the 1–2 h time interval compared to the 0–1 h time interval (151.5 AU vs. 186.0 AU, *p* = 0.018) (Table 2, Figure 2).

Flap tissue perfusion also differed across time intervals in terms of hemoglobin oxygen saturation (*p* < 0.001), with lower hemoglobin oxygen saturation in the 2–3 h time interval compared to the 1–2 h time interval (76.0 AU vs. 78.0 AU, *p* = 0.003) (Table 2, Figure 2).

### 3.3. Association of Flap Tissue Perfusion Measurement Values and Blood Pressure Values

SBP was associated with blood flow, showing a negative correlation between the absolute values of SBP and blood flow (−0.114, *p* = 0.016) and a positive correlation between the relative values of SBP and blood flow (0.153, *p* = 0.001) (Table 3). Neither correlation persisted upon controlling for norepinephrine, propofol, or sulfentanyl (all *p* > 0.05).

**Figure 3:**
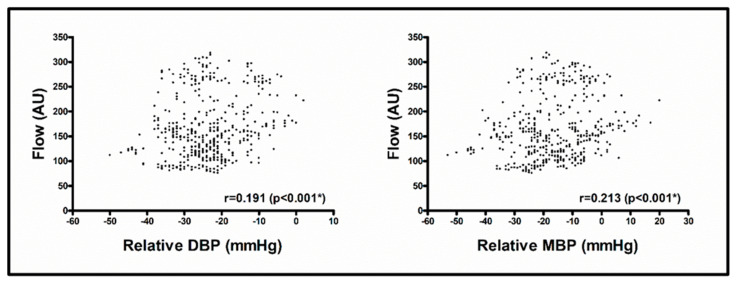
Association of flap perfusion measurement values with blood pressure values. Data shown as scatter plot for blood flow and relative DBP (diastolic blood pressure) (**left**) and blood flow and relative MBP (mean arterial blood pressure) (**right**); r = Spearman correlation coefficient (with *p*-value); * *p* < 0.05 for calculation of partial Spearman correlation with controlling for norepinephrine, propofol or sulfentanyl; abbreviations: AU = arbitrary units, DBP = diastolic blood pressure, MBP = mean arterial blood pressure.

DBP was also associated with blood flow and hemoglobin oxygen saturation, with a positive correlation between the relative values of DBP and blood flow (0.191, *p* < 0.001) and a negative correlation between the relative values of DBP and hemoglobin oxygen saturation (−0.138, *p* = 0.003) (Table 3, Figure 3). The correlation between the relative values of DBP and blood flow persisted upon controlling for norepinephrine, propofol, or sulfentanyl (all *p* < 0.05).

MBP was associated with blood flow, showing a positive correlation between the relative values of MBP and blood flow (0.213, *p* < 0.001) (Table 3, Figure 3). The correlation persisted upon controlling for norepinephrine, propofol, or sulfentanyl (all *p* < 0.05).

## 4. Discussion

This pilot study investigated the relationship between RFFF tissue perfusion and systemic blood pressure in postoperative flap monitoring after microvascular head and neck reconstruction, as the O2C analysis system used for postoperative flap monitoring relies on the measurement of flap tissue perfusion in comparison to predefined thresholds and simultaneously flap tissue perfusion might be influenced by systemic blood pressure [5,7,8].

Postoperative flap monitoring is considered essential to timely detect vascular flap compromise in microvascular head and neck reconstruction [5,6,7,8]. In this context, the O2C analysis system, which is based on the measurement of flap tissue perfusion in relation to predefined absolute thresholds for blood flow and hemoglobin oxygen saturation, is routinely used [5,7,8]. However, the comparison of measured flap tissue perfusion to predefined absolute thresholds, indicative of vascular flap compromise, may be confounded by the potential influence of systemic blood pressure on flap tissue perfusion, as systemic blood pressure is an important determinant of general tissue perfusion and likely also of flap tissue perfusion [5,8,12,13,14]. Patients undergoing microvascular head and neck reconstruction frequently exhibit postoperative alterations in systemic blood pressure, and the microvascular flap tissue is particularly sensitive to alterations in systemic blood pressure due to denervation [20,21]. However, the influence of systemic blood pressure on flap tissue perfusion is still unknown, as previous studies have included patients with different flap types and lacked an assessment of the specific parameters of flap tissue perfusion, such as blood flow and hemoglobin oxygen saturation [16,17].

Due to the low rates of flap revision and failure in microvascular head and neck reconstruction, this study investigated the influence of systemic blood pressure on flap tissue perfusion rather than directly evaluating its potential confounding effect on the validity of the thresholds of blood flow and hemoglobin oxygen saturation in postoperative flap monitoring using the O2C analysis system [3,5,8]. In addition, this study utilized attached surface probes for the O2C analysis system, as they facilitate a continuous measurement of flap tissue perfusion and are less susceptible to confounding factors such as surface probe dislocation and pressure alterations, both of which contribute to variations in flap tissue perfusion measurements [22,23,24].

This study demonstrated that flap tissue perfusion, in terms of blood flow and hemoglobin oxygen saturation, generally did not differ between the time intervals of the measurement in the early postoperative period.

This observation is consistent with the results of a previous study that also examined flap tissue perfusion using the O2C analysis system in patients undergoing microvascular head and neck reconstruction with RFFFs and revealed that blood flow and hemoglobin oxygen saturation did not differ between an intraoperative measurement time point and a measurement time point on the first postoperative day [5]. However, the previous study can only serve as an orientation, as its two measurement timepoints differed from the measurement time interval of the present study [5]. In addition, the previous study used unattached surface probes with the O2C analysis system that measured flap tissue perfusion at 2 and 8 mm tissue depths, which hampers a comparison between the two studies, as flap tissue perfusion, in terms of blood flow and hemoglobin oxygen saturation, differs between tissue depths in the microvascular free flaps used for head and neck reconstruction [11,25]. Furthermore, the dependence of hemoglobin oxygen saturation on blood flow revealed in a previous study may explain the observed decreasing trend, first in the blood flow between the two time intervals of 1–2 h and 0–1 h and subsequently in the hemoglobin oxygen saturation between the two time intervals of 2–3 h and 1–2 h postoperatively [5]. In general, addressing the physiology of microvascular free flap perfusion, which is still largely unknown, RFFFs show constant flap tissue perfusion in terms of blood flow and hemoglobin oxygen saturation in the early postoperative period [15].

This study also demonstrated that RFFF tissue perfusion was associated with systemic blood pressure, in terms of a positive correlation between blood flow and relative DBP and MBP.

The association between RFFF tissue perfusion and systemic blood pressure might reflect the role of the vessel perfusion pressure gradient as a determinant of general tissue perfusion, in addition to vessel diameter, vessel length, and blood viscosity, with MBP presumably reflecting the vessel perfusion pressure gradient at most [12,14,26]. Additionally, assuming constant vessel length and blood viscosity, denervation of the microvascular free flap with a loss of neural adrenergic vascular tone control after flap harvest may increase the influence of systemic blood pressure on flap tissue perfusion [12,14,20,27,28]. However, it should be considered that various other control mechanisms also influence flap vessel diameter and that the correlations between DBP and MBP and blood flow were quantitatively low [29,30]. Nevertheless, with regard to the correlations between DBP and MBP, it should be noted that the correlations persisted upon adjustment for norepinephrine, propofol, or sulfentanyl, which influence general tissue perfusion and probably also flap tissue perfusion [31,32]. In general, the observation that only the relative and not the absolute blood pressure values were associated with flap tissue perfusion, in terms of blood flow, may be due to the individual physiology of each patient being more reflected by the relative blood pressure values, which account for the normally prevailing baseline blood pressure values [33,34].

The limitations of this study are the small number of patients included and the short time interval of the postoperative period studied. However, all patients included in the study underwent reconstruction with the same flap type, which improves the comparability of flap tissue perfusion measurements, and the time interval for postoperative flap tissue perfusion measurement used for analysis in this study was limited by the data availability for systemic blood pressure measured invasively with an arterial catheter, as well as for norepinephrine, propofol, or sulfentanyl, which affect flap tissue perfusion [5,8,31,32,35]. Furthermore, interpatient differences in the factors that may potentially affect flap perfusion, such as vessel length and vessel diameter, as well as flap location, cannot be ruled out.

This study was conducted to evaluate the relationship between RFFF tissue perfusion and systemic blood pressure in the context of early postoperative flap monitoring with the O2C analysis system using attached surface probes, which have already been shown to be an option for flap perfusion monitoring in microvascular head and neck reconstruction [11,25]. However, as the O2C analysis system relies on the measurement of flap tissue perfusion in relation to predefined absolute thresholds for blood flow and hemoglobin oxygen saturation, an influence of systemic blood pressure on flap tissue perfusion might confound postoperative flap monitoring [5,8]. The study found that flap tissue perfusion in terms of blood flow and hemoglobin oxygen saturation generally remains constant in the early postoperative period but correlates with systemic blood pressure in terms of blood flow and relative blood pressure values. This potentially confounding effect of systemic blood pressure should be considered in postoperative flap monitoring after microvascular head and neck reconstruction with regard to the absolute thresholds for blood flow. In terms of clinical implications, the results suggest that systemic blood pressure during flap tissue perfusion measurement should be constant for all measurement timepoints to preserve the accuracy of the O2C analysis system. Further prospective studies with a larger study cohort and a more extended postoperative study period are needed to confirm the results.

## 5. Conclusions

This study showed that early postoperative tissue perfusion of RFFFs, in terms of blood flow and hemoglobin oxygen saturation, generally remains constant, but correlates with systemic blood pressure in terms of blood flow and relative blood pressure values. This correlation should be considered in postoperative flap monitoring of RFFFs, especially with regard to the absolute thresholds for blood flow. To prevent or at least minimize any confounding effects, systemic blood pressure should be kept constant at all measurement timepoints during flap tissue perfusion measurement. Further studies on this topic should include a larger study cohort and a longer study period.

## Figures and Tables

**Table 1:** Clinical characteristics of the study population.

Variable	(n = 14)
Sex (n)	
Male	5 (35.7%)
Female	9 (64.3%)
Age (years)	68.5 (28.0)
BMI (kg/m^2^)	26.0 (10.0)
ASA (n)	
1	0 (0.0%)
2	5 (35.7%)
3	9 (64.3%)
4	0 (0.0%)
Flap location (n)	
intraoral	
tongue	4 (28.6%)
mouth floor	2 (14.3%)
mandible	3 (21.4%)
cheek	1 (7.1%)
extraoral	
buccal	2 (14.3%)
infraorbital	1 (7.1%)
orbital	1 (7.1%)
Arterial anastomosis recipient vessel (n)	
facial artery	8 (57.1%)
lingual artery	1 (7.1%)
superior thyroid artery	5 (35.7%)
Venous anastomosis recipient vessel (n)	
internal jugular vein	9 (64.3%)
internal jugular vein + other vein	3 (21.4%)
other vein	2 (14.3%)
Surgery duration (min)	508.5 (151.0)
Flap ischemia duration (min)	81.5 (25.0)

Parameters are indicated as numbers (with percentage) for categorical data (sex, ASA, flap location, arterial anastomosis recipient vessels, and venous anastomosis recipient vessel) or median (with interquartile range) for metric data (age, BMI, surgery duration, and flap ischemia duration); internal jugular vein + other = internal jugular vein and anterior jugular vein or internal jugular vein and vena comitans of hypoglossal nerve; other vein = facial vein or retromandibular vein; abbreviations: BMI = body mass index, ASA = American Society of Anesthesiologists score.

**Table 2:** Comparison of flap tissue perfusion over time.

Time Interval (Hours)	Perfusion Parameters			
	Blood Flow (AU)	*p*-Value	Hemoglobin Oxygen Saturation (%)	*p*-Value
**0–1**	186.0 (76.0)	-	78.0 (14.0)	-
**1–2**	151.5 (70.0)	**0.018**	78.0 (10.0)	1.000
**2–3**	148.0 (63.0)	1.000	76.0 (11.0)	**0.003**
**3–4**	136.5 (62.0)	1.000	76.0 (9.0)	1.000
**4–5**	150.0 (78.0)	0.820	74.5 (12.0)	1.000
**5–6**	142.0 (75.0)	1.000	77.0 (13.0)	1.000
**6–7**	149.0 (72.0)	1.000	76.0 (14.0)	1.000
**7–8**	144.5 (102.0)	1.000	73.5 (13.0)	1.000

Perfusion measurement values are indicated as median (with interquartile range) for each measurement time interval (0–1 h, 1–2 h, 2–3 h, 3–4 h, 4–5 h, 5–6 h, 6–7 h, and 7–8 h) separately for blood flow and hemoglobin oxygen saturation; *p*-values corresponding to testing for differences between measurement time intervals with Dunn-Bonferroni post hoc test (1–2 h vs. 0–1 h, 2–3 h vs. 1–2 h, 3–4 h vs. 2–3 h, 4–5 h vs. 3–4 h, 5–6 h vs. 4–5 h, 6–7 h vs. 5–6 h, and 7–8 h vs. 6–7 h) after Friedman test (blood flow *p* < 0.001; hemoglobin oxygen saturation *p* < 0.001); significant *p*-values are bold; abbreviations: AU = arbitrary units.

**Table 3:** Association of flap tissue perfusion with blood pressure values.

Blood Pressure Parameters	Perfusion Parameters			
	Blood Flow (AU)	*p*-Value	Hemoglobin Oxygen Saturation (%)	*p*-Value
**SBP**				
**Absolute values**	−0.114	**0.016**	−0.002	0.974
**Relative values**	0.153	**0.001**	0.025	0.599
**DBP**				
**Absolute values**	−0.014	0.768	−0.053	0.266
**Relative values**	0.191	<0.001 *	−0.138	**0.003**
**MBP**				
**Absolute values**	−0.062	0.191	−0.036	0.448
**Relative values**	0.213	<0.001 *	−0.046	0.333

Data presented as Spearman correlation coefficient (with *p*-value) calculated between blood pressure parameters (systolic blood pressure [SBP], diastolic blood pressure [DBP], and mean arterial blood pressure [MBP]) and perfusion parameters (blood flow and hemoglobin oxygen saturation); significant *p*-values are bold; * *p* < 0.05 for calculation of partial Spearman correlation with controlling for norepinephrine, propofol, or sulfentanyl; abbreviations: AU = arbitrary units, SBP = systolic blood pressure, DPB = diastolic blood pressure, MBP = mean arterial blood pressure.

## Data Availability

The raw data supporting the conclusions of this article will be made available by the authors on request.

## Abbreviations

The following abbreviations are used in this manuscript:

RFFF	Radial free forearm flap
O2C	Oxygen-2-See
SBP	Systolic blood pressure
DBP	Diastolic blood pressure
MBP	Mean arterial blood pressure
